# Supplementary material for: Molecular and functional profiling identifies therapeutically targetable vulnerabilities in plasmablastic lymphoma
Source: Nat Commun. 2021 Aug 31;12:5183. doi: 10.1038/s41467-021-25405-w (PMC8408158; doi:10.1038/s41467-021-25405-w)
Supplement: Supplementary file 1 — Supplementary Information [file 41467_2021_25405_MOESM1_ESM.pdf]

Supplementary Information for

Molecular and functional profiling identifies therapeutically targetable vulnerabilities in plasmablastic lymphoma

Fabian Frontzek[1], Annette M. Staiger[2,3], Myroslav Zapukhlyak[1], Wendan Xu[1], Irina Bonzheim[4], Vanessa Borgmann[4], Philip Sander[4], Maria Joao Baptista[5], Jan-Niklas Heming[1], Philipp Berning[1], Ramona Wullenkord[1], Tabea Erdmann[1], Mathias Lutz[1], Pia Veratti[6], Sophia Ehrenfeld[7], Kirsty Wienand[8], Heike Horn[2,3], John R. Goodlad[9], Matthew R. Wilson[10], Ioannis Anagnostopoulos[11,12], Mario Lamping[12], Eva Gonzalez-Barca[13], Fina Climent[14], Antonio Salar[15], Josep Castellvi[16], Pau Abrisqueta[17], Javier Menarguez[18], Teresa Aldamiz[19], Julia Richter[20], Wolfram Klapper[20], Alexandar Tzankov[21], Stefan Dirnhofer[21], Andreas Rosenwald[11], José Luis Mate[22], Gustavo Tapia[22], Peter Lenz[23], Cornelius Miething[7,24,25], Wolfgang Hartmann[26], Björn Chapuy[8], Falko Fend[4], German Ott[3], José-Tomas Navarro[5], Michael Grau#[1], Georg Lenz#*[1]

# Georg Lenz and Michael Grau contributed equally.

1 Department of Medicine A, Department of Hematology, Oncology and Pneumology, University Hospital Münster, Münster, Germany
2 Dr. Margarete Fischer-Bosch Institute of Clinical Pharmacology, Stuttgart and University of Tuebingen, Germany
3 Department of Clinical Pathology, Robert Bosch Hospital, Stuttgart, Germany
4 Institute of Pathology and Neuropathology, Eberhard Karls University of Tübingen-Comprehensive Cancer Center, University Hospital Tübingen, Tübingen, Germany
5 Department of Hematology, ICO-Hospital Germans Trias i Pujol, Josep Carreras Leukaemia Research Institute (IJC), Universitat Autònoma de Barcelona, Badalona, Spain
6 Department of Psychiatry and Psychotherapy, University Hospital Freiburg, Freiburg, Germany
7 Department of Medicine I, Medical Center, Faculty of Medicine, University of Freiburg, Freiburg, Germany
8 Department of Hematology and Medical Oncology, University Medical Center Göttingen, Göttingen, Germany
9 Department of Pathology, Queen Elizabeth University Hospital, Glasgow, United Kingdom
10 Department of Haematology, Beatson West of Scotland Cancer Centre, Glasgow, United Kingdom
11 Institute of Pathology, University of Würzburg, Würzburg, Germany
12 Charité Comprehensive Cancer Center, Charité - Universitätsmedizin Berlin, Corporate Member of Freie Universität Berlin, Humboldt-Universität zu Berlin and Berlin Institute of Health, Berlin, Germany
13 Department of Hematology. ICO-Hospital Duran i Reynals, Universitat de Barcelona, L'Hospitalet de Llobregat, Spain
14 Department of Pathology. Hospital Universitari de Bellvitge-IDIBELL, L'Hospitalet de Llobregat, Spain
15 Department of Hematology. Hospital del Mar, Barcelona, Spain
16 Department of Pathology. Hospital Universitari Vall d'Hebron, Barcelona, Spain
17 Department of Hematology. Hospital Universitari Vall d'Hebron, Barcelona, Spain
18 Department of Pathology. Hospital Gregorio Marañón, Madrid, Spain
19 Department of Infectious Diseases, Hospital Gregorio Marañón, Madrid, Spain
20 Division of Hematophathology, Christian-Albrechts-University, Kiel, Germany
21 Institute of Medical Genetics and Pathology, University Hospital Basel, Basel, Switzerland
22 Department of Pathology, Hospital Germans Trias i Pujol, Institut Germans Trias i Pujol (IGTP), Universitat Autònoma de Barcelona, Badalona, Spain
23 Department of Physics, University of Marburg, Marburg, Germany
24 German Cancer Consortium (DKTK), Partner Site Freiburg, Freiburg, Germany.
25 German Cancer Research Center (DKFZ), Heidelberg, Germany.
26 Division of Translational Pathology, Gerhard-Domagk-Institute of Pathology, University Hospital Münster, Münster, Germany

* Corresponding author
Georg Lenz, M.D.
Department of Medicine A of Hematology, Oncology, and Pneumology
48149 Münster, Germany
Phone: +49 251 83 47587
Fax: +49 251 83 47588
Email: Georg.Lenz@ukmuenster.de

Table of Contents

Supplementary Figures **1**
Supplementary Fig. 1: Summary of performed analyses of primary PBL samples 1
Supplementary Fig. 2: MYC FISH in primary PBL samples 2
Supplementary Fig. 3: Schematic overview of the analysis pipeline 3
Supplementary Fig. 4: Effective Coverage of WES data 4
Supplementary Fig. 5: PBLs have a high tumor mutational burden (TMB) 5
Supplementary Fig. 6: Landscape of somatic mutations in PBL determined by WES 6
Supplementary Fig. 7: Mutation diagrams of selected cancer candidate genes (CCGs) 7
Supplementary Fig. 8: Detected somatic mutations in PBL cases with a matched normal control 8
Supplementary Fig. 9: Detected somatic mutations in PBL cases without a matched normal control 9
Supplementary Fig. 10: Identification of targets for the treatment of PBL 10
Supplementary Fig. 11: Impact of DNA quality and tumor cell content on total mutational burden (TMB) 11

Supplementary Tables **12**
Supplementary Table 1: Baseline characteristics of PBL patients included in survival analysis 12
Supplementary Table 2: Used antibodies for immunohistochemistry 13
Supplementary Table 3: Availabilities for utilized methods, tools, databases and software 14
Supplementary Table 4: List of used primers 16

Supplementary Figures

Supplementary Fig. 1: Summary of performed analyses of primary PBL samples

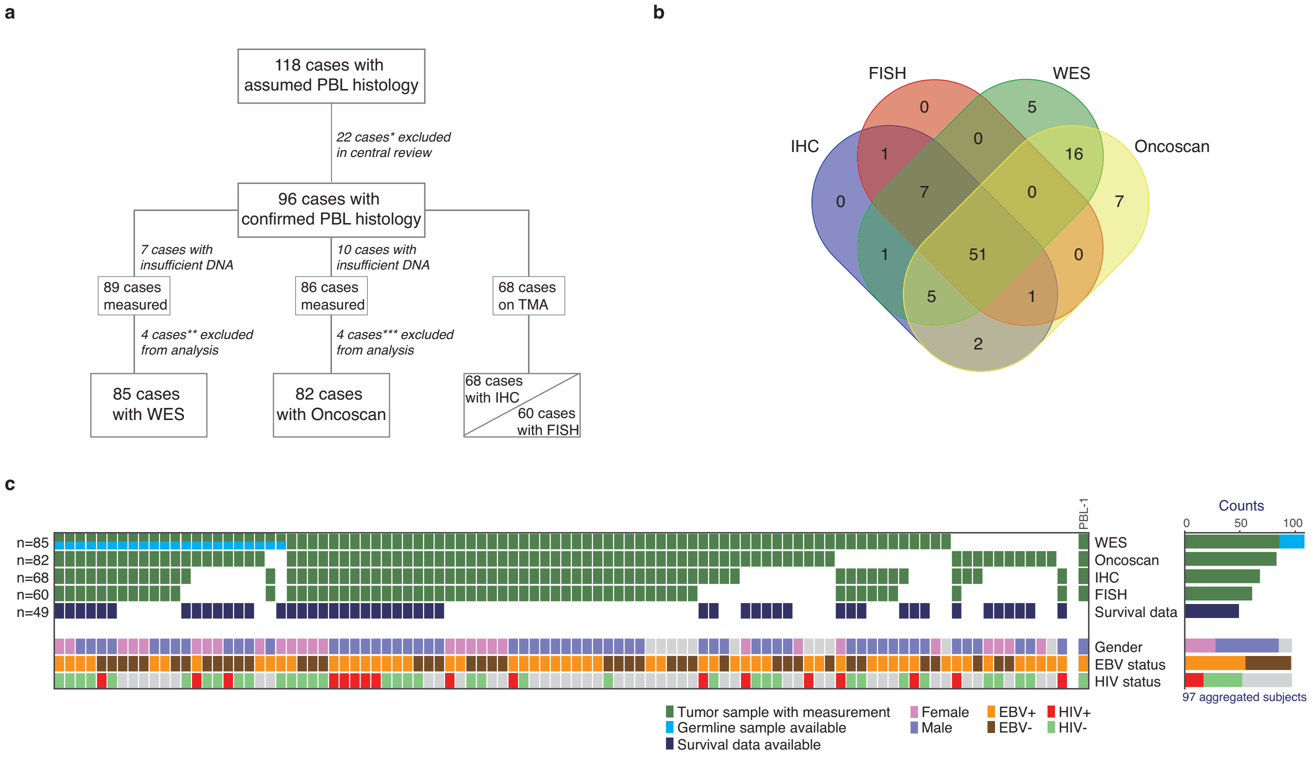

(a) Flow diagram showing selection and subsequent analysis of PBL samples. (*)22 cases were initially excluded in the central pathology review due to diagnosis of plasma cell neoplasm (n=12), diffuse large B-cell lymphoma (DLBCL; n=1), necrotic/ apoptotic lymphoma (n=2), ALK+ DLBCL (n=4), pleural effusion lymphoma (n=3). (**)Three samples were excluded from WES analysis due to low effective coverage and one further sample due to hypermutation. (***)Four samples were excluded from Oncoscan analysis due to quality control of ASCAT. 68 samples were suitable for TMA construction and further analyzed by FISH and immunohistochemistry (IHC). **(b)** Venn diagram illustrating intersections of how many PBL samples have been analyzed by IHC, FISH, WES, and Oncoscan. (c) WES, Oncoscan analysis, FISH, and IHC were performed in the indicated numbers of PBL samples. Additionally, presence of survival data, annotated gender, HIV status, and measured EBV status are indicated for each PBL sample per column and for the cell line PBL-1. The bar graph on the right side indicates the total amount of measured samples.

Supplementary Fig. 2: MYC FISH in primary PBL samples

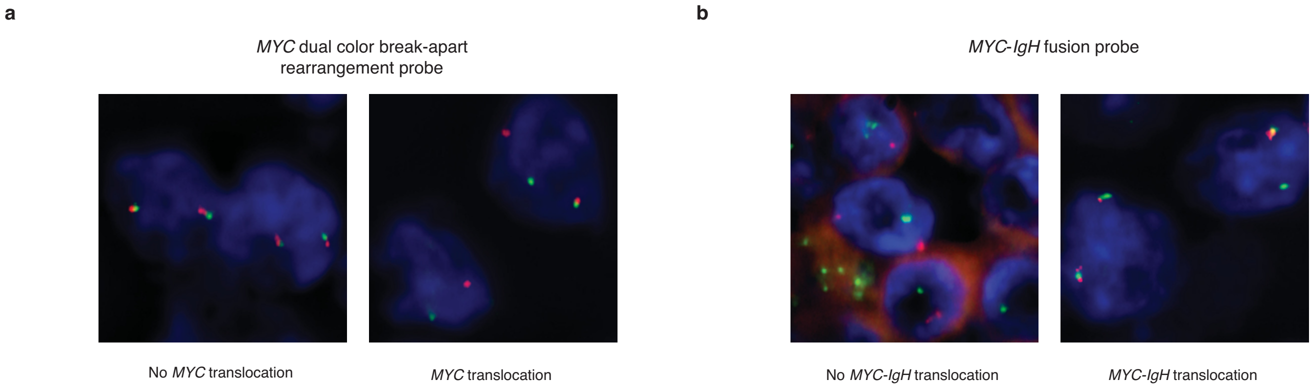

Representative fluorescence microscopy images showing a *MYC* translocated PBL case **(a)** using a *MYC* dual-color break apart probe (100x zoom). In case of a *MYC* translocation, the normally co-localized red and green fluorescence signals (left) are separated in at least 15% of analyzed cells (right). **(b)** Red and green fluorescence signals of a *MYC-IgH* fusion probe mark the gene loci of *MYC* and *IgH,* respectively (left). In case of a *MYC-IgH* translocation, signals are fused in at least 9% of analyzed cells as shown (right). At least 100 intact nuclei per case were evaluated.

Supplementary Fig. 3: Schematic overview of the analysis pipeline

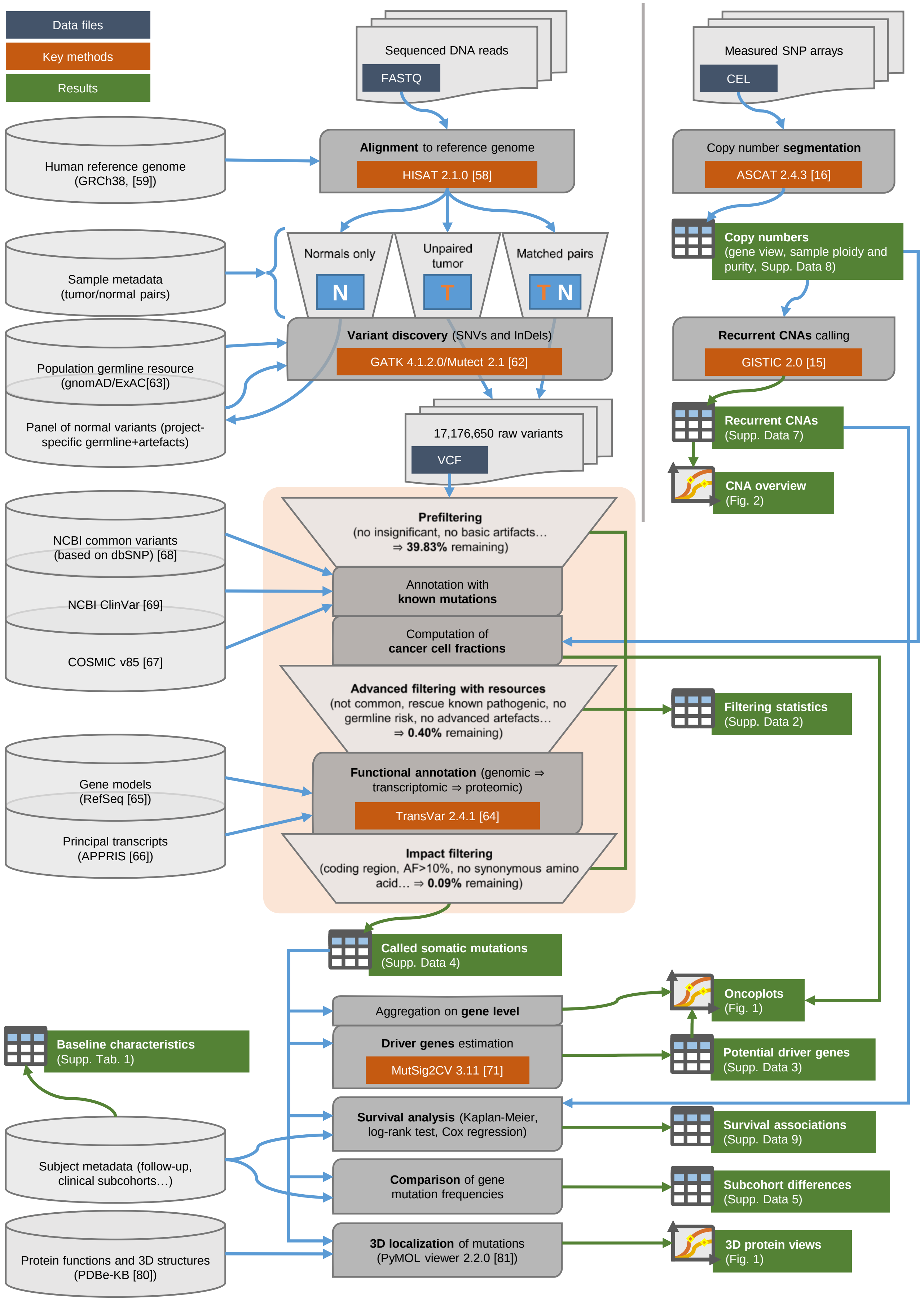

Utilized resource databases and metadata are depicted as grey discs, primary source data are shown in dark blue, applied key methods in orange, and resulting figures and tables in green color.

Supplementary Fig. 4: Effective Coverage of WES data

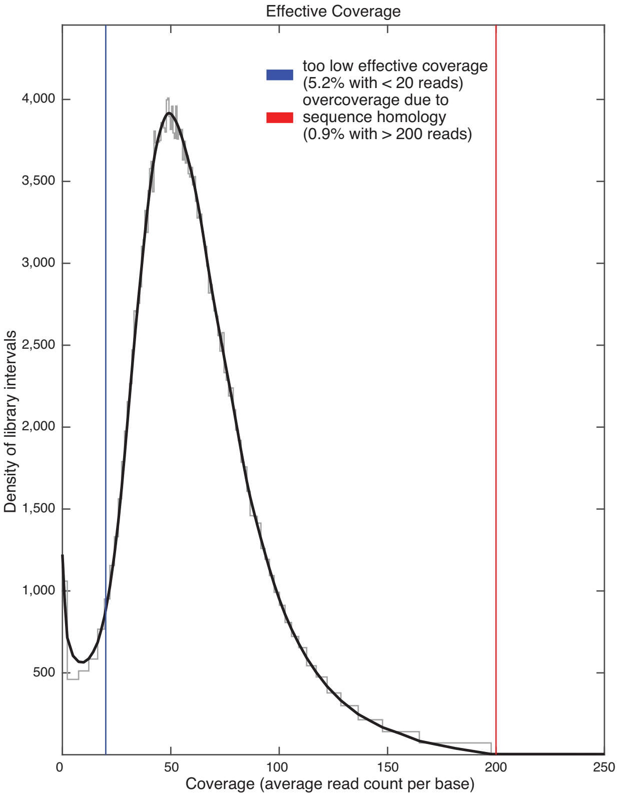

The effective coverage (average reads per base) for measured genomic intervals after applying all read filters. Limits for too low (blue) or too high coverage (red) due to sequence homologies are indicated.

Supplementary Fig. 5: PBLs have a high tumor mutational burden (TMB)

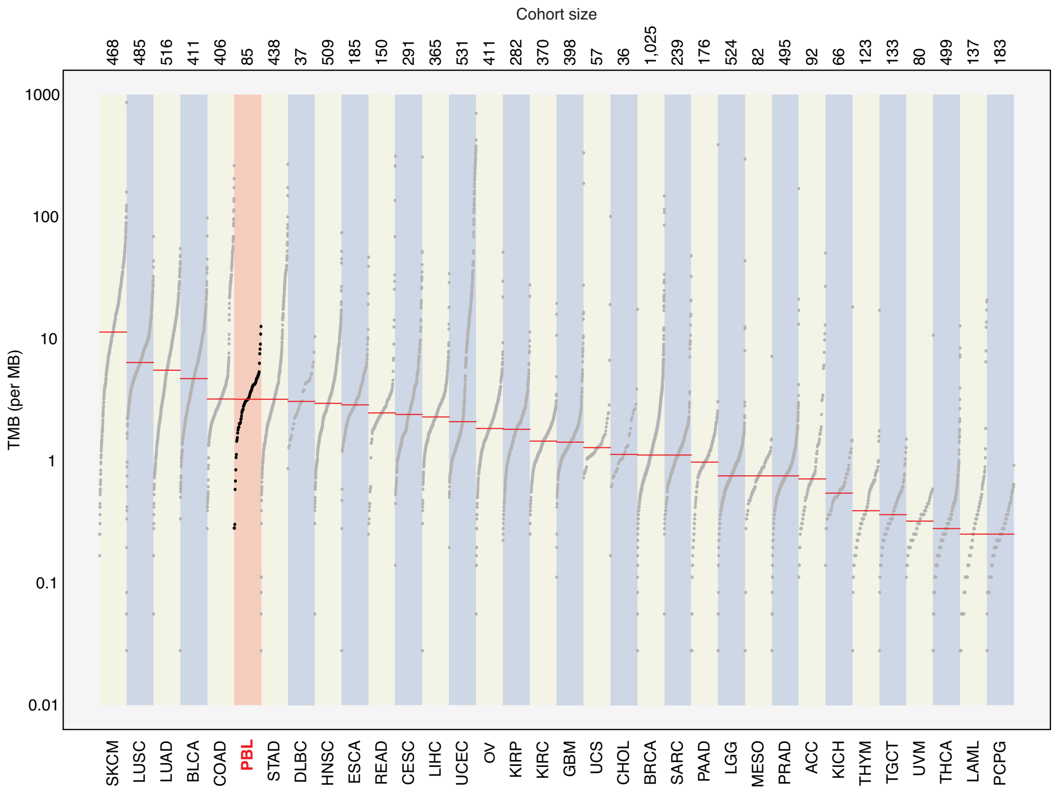

TMB per megabase (MB) for different cancer entities. PBLs have a high median TMB of 3.20/MB compared to other tumors. Data with corresponding TMB were provided by the TCGA database for different cancers and the plot was created using maftool v2.7.41[78].

TCGA study abbreviations: ACC: adrenocortical carcinoma, BLCA: bladder urothelial carcinoma, BRCA: breast invasive carcinoma, CESC: cervical squamous cell carcinoma and endocervical adenocarcinoma, CHOL: cholangiocarcinoma, COAD: colon adenocarcinoma, DLBCL: diffuse large B-cell lymphoma, ESCA: esophageal carcinoma, GBM: glioblastoma multiforme, HNSC: head and neck squamous cell carcinoma, KICH: kidney chromophobe, KIRC: kidney renal clear cell carcinoma, KIRP: kidney renal papillary cell carcinoma, LAML: acute myeloid leukemia, LGG: brain low grade glioma, LIHC: liver hepatocellular carcinoma, LUAD: lung adenocarcinoma, LUSC: lung squamous cell carcinoma, MESO: mesothelioma, OV: ovarian serous cystadenocarcinoma, PAAD: pancreatic adenocarcinoma, PCPG: pheochromocytoma and paraganglioma, PRAD: prostate adenocarcinoma, READ: rectum adenocarcinoma, SARC: sarcoma, SKCM: skin cutaneous melanoma, STAD: stomach adenocarcinoma, TGCT: testicular germ cell tumors, THCA: thyroid carcinoma, THYM: thymoma, UCEC: uterine corpus endometrial carcinoma, UCS: uterine carcinosarcoma, UVM: uveal melanoma.

Supplementary Fig. 6: Landscape of somatic mutations in PBL determined by WES

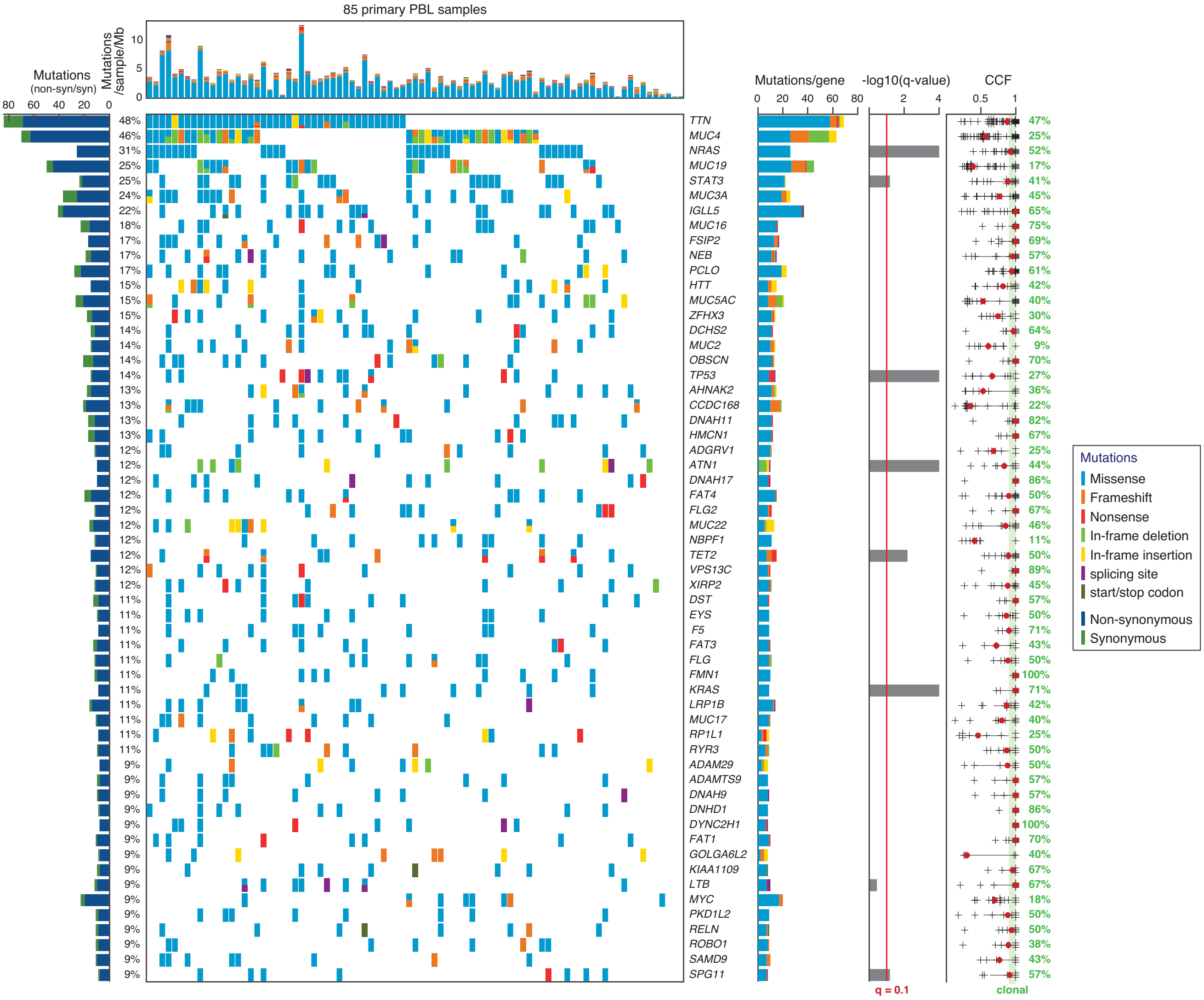

All called non-synonymous mutations (in genes with cohort frequency≥9%) are color-coded and shown for each sample per column and ranked by cohort frequency. Samples are ordered by waterfall sorting based on binary gene mutation status. The bar graph on the left shows the ratio of non-synonymous (blue) and synonymous (green) mutations per gene. At the top, the TMB per sample is depicted (mutations/sample/Mb). At the right, different types of mutation and q-values (M2CV) are shown per gene. For each gene, CCF was estimated for samples with corresponding copy number measurement (median in red). The percentage of samples with clonal mutations is indicated per gene.

Supplementary Fig. 7: Mutation diagrams of selected cancer candidate genes (CCGs)

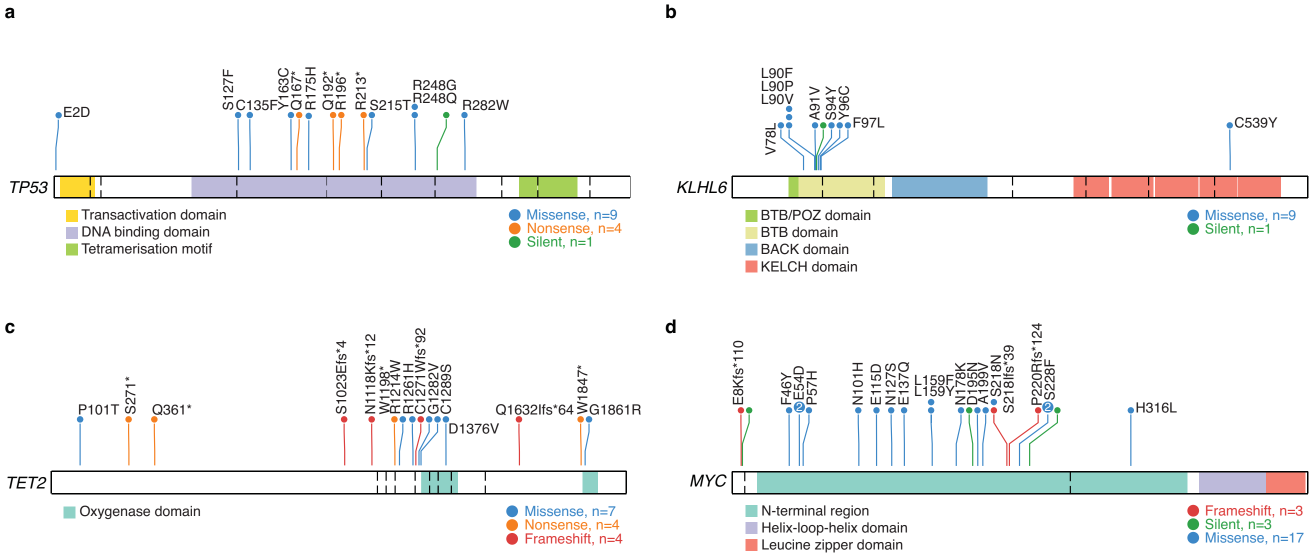

The distribution of mutations on protein level for the selected CCGs **(a)** *TP53* (NM_001126112), **(b)** *KLHL6* (NM_130446), **(c)** *TET2* (NM_001127208), and **(d)** *MYC* (NM_002467). Exon boundaries are indicated using dashed lines.

Supplementary Fig. 8: Detected somatic mutations in PBL cases with a matched normal control

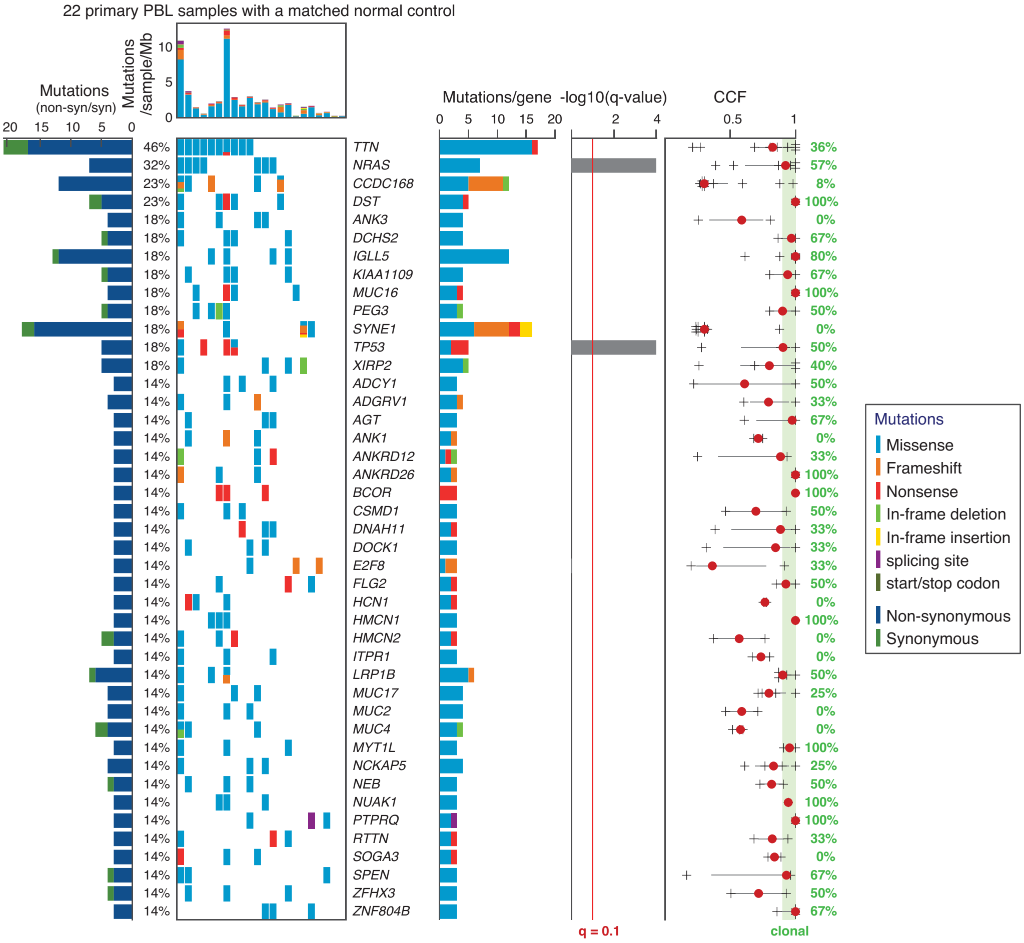

For the subcohort of PBL cases having a matched normal control (n=22), all called somatic mutations (cohort frequency≥10%) are shown as in Supplementary Fig. 6.

Supplementary Fig. 9: Detected somatic mutations in PBL cases without a matched normal control

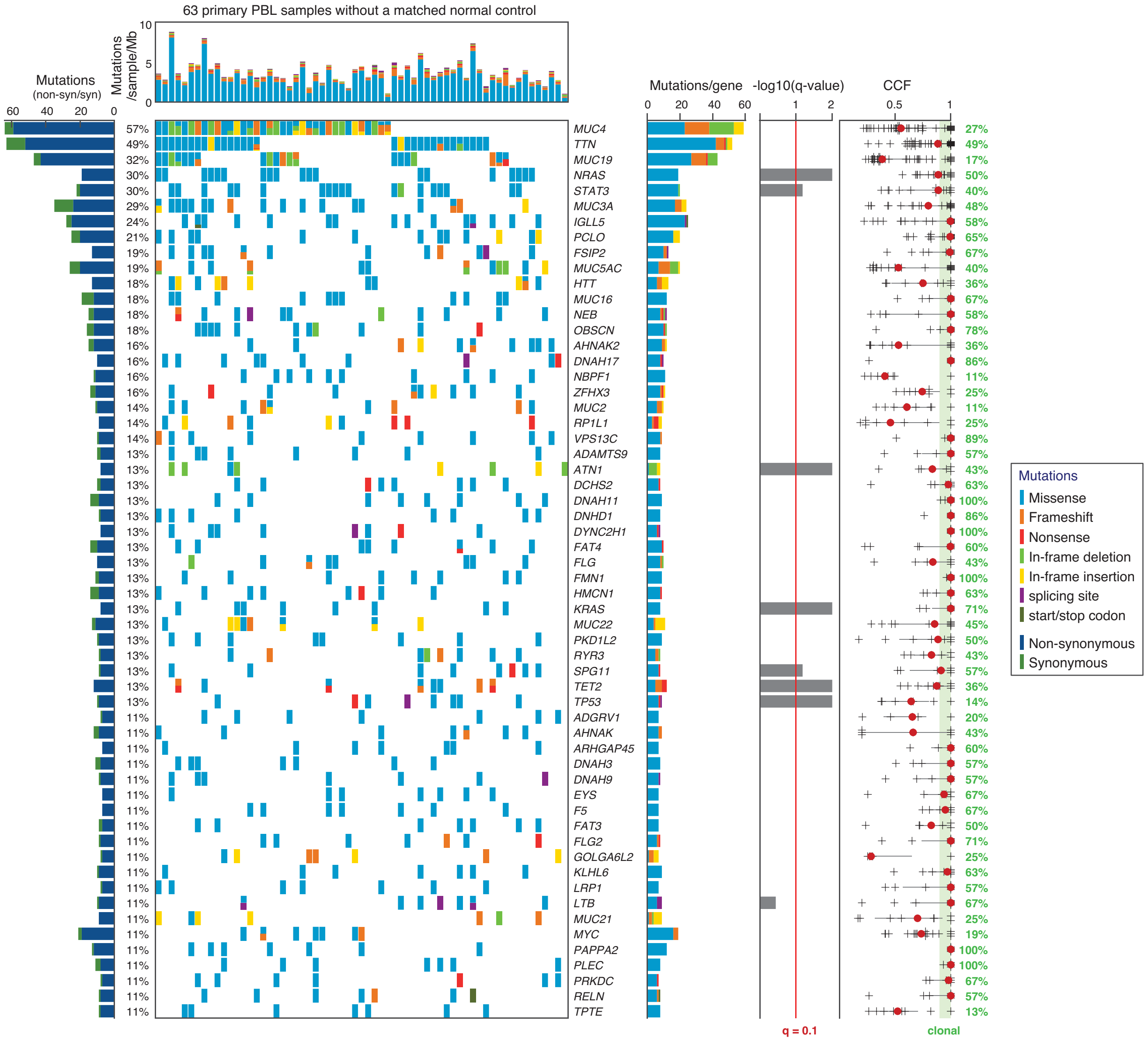

For the subcohort of PBL cases without matched normal control (n=63), all called somatic mutations (cohort frequency≥10%) are shown as in Supplementary Fig. 6.

Supplementary Fig. 10: Identification of targets for the treatment of PBL

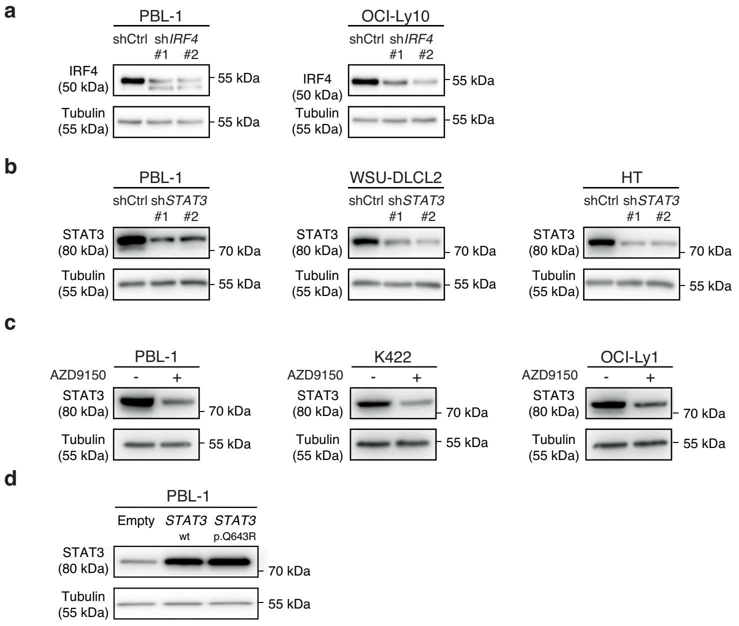

(a) IRF4 protein expression determined by Western blotting in PBL-1 and OCI-Ly10 cells following shRNA *IRF4*#1 and #2 induced knockdown for four days compared to control shRNA. Representative results are shown of three independent experiments. **(b)** STAT3 protein expression determined by Western blotting in PBL-1, HT, and WSU-DLCL2 cells following shRNA *STAT3*#1 and #2 induced knockdown for four days compared to control shRNA. Representative results are shown of two independent experiments. **(c)** STAT3 protein expression determined by Western blotting in PBL-1, OCI-Ly1, and K422 cells following antisense control (-) or AZD9150 (+) [25 μM] treatment for 24h. Representative results are shown of three independent experiments. **(d)** Levels of STAT3 determined by Western blotting in PBL-1 cells following transduction of control (empty), wildtype (wt) *STAT3,* and p.Q643R *STAT3* cDNA. Representative results are shown of three independent experiments. Source data are provided as a Source Data file.

Supplementary Fig. 11: Impact of DNA quality and tumor cell content on total mutational burden (TMB)

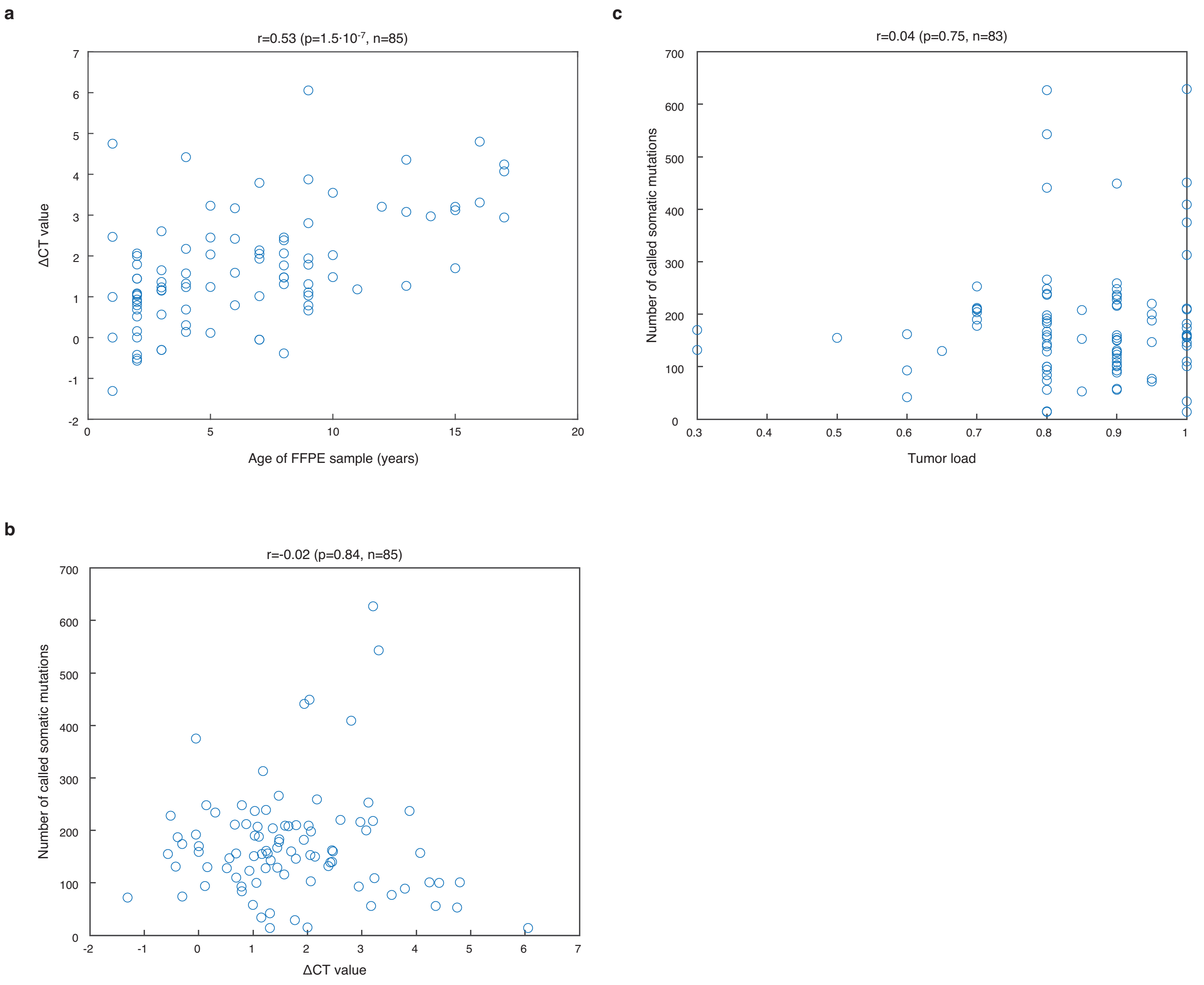

Scatter plots depicting correlation between **(a)** measured ΔCt values of DNA and age of corresponding FFPE specimen (year) ($r=0.53$, $p=1.5*10^{-7}$), **(b)** number of called somatic mutations per sample and corresponding ΔCt value ($r=-0.02$, $p=0.84$), and **(c)** number of called somatic mutations per sample and corresponding microscopically determined tumor cell content (%) ($r=0.04$, $p=0.75$). Source data are provided as a Source Data file.

Supplementary Tables

Supplementary Table 1: Baseline characteristics of PBL patients included in survival analysis

Characteristic		Overall
**Number of patients**		49
**Age at diagnosis in years** (median) (min-max range)		62<br>(23 - 89)
**Sex**	Female	15 (31%)
	Male	34 (69%)
**IPI**	Low	13 (32%)
	Intermediate	19 (46%)
	High	9 (22%)
	Unknown	8
**HIV**	Negative	31 (69%)
	Positive	14 (31%)
	Unknown	4
**EBV**	Negative	21 (43%)
	Positive	28 (57%)
	Unknown	0
**Extranodal involvement**	Yes	37 (82%)
	No	8 (18%)
	Unknown	4
**Bulk**	Yes	10 (23%)
	No	33 (77%)
	Unknown	6
**Chemotherapy**	CHOP-like	37 (82%)
	None or other	8 (18%)
	Unknown	4
**Radiotherapy in first-line therapy**	Yes	16 (37%)
	No	27 (63%)
	Unknown	6
**Follow-up in months** (median) (min-max range in days)		21.5<br>(6 - 6,014)

Abbreviations: min: minimum; max: maximum; IPI: International Prognostic Index; HIV: human immunodeficiency virus; EBV: Epstein-Barr virus; CHOP: cyclophosphamide, hydroxydaunorubicin, vincristine, prednisone. For each category, 100% correspond to cases with available data.

Supplementary Table 2: Used antibodies for immunohistochemistry

Antibody	Clone	Source	Detection System	Dilution	Cutoff for positivity
CD56	MRQ-42	Cell Marque	HRP Polymer System	1 to 400	30%
CD138	B-A38	Cell Marque	HRP Polymer System	1 to 300	30%
CD30	Ber-H2	Bio SB	HRP Polymer System	1 to 25	30%
PDL1	ZR3	ZETA Corp	HRP Polymer System	1 to 300	30%
CD3	2GV6	Roche	Ventana Benchmark	prediluted	30%
CD20	L26	Roche	Ventana Benchmark	prediluted	30%
CD38	SP149	Roche	Ventana Benchmark	prediluted	30%
IRF4/MUM1	MRQ-43	Roche	Ventana Benchmark	prediluted	30%
ALK1	ALK01	Roche	Ventana Benchmark	prediluted	30%
Blimp1	PRDM1	Abcam	Ventana Benchmark Ultra	1 to 100	Recorded in increments of 10%
Ki67	30-9	Roche	Ventana Benchmark Ultra	prediluted	Recorded in increments of 10%
HHV8 Lana	13B10	Roche	Ventana Benchmark Ultra	prediluted	Positive or negative
MYC	Y69	Roche	Ventana Benchmark Ultra	prediluted	40%
CD19	BT51E	Leica Biosystems	Bond polymer refine detection kit	1 to 50	30%
PAX5	24/PAX-5	BD Transduction	Bond polymer refine detection kit	1 to 10	30%
EBER	EBER Oligo-nucleotide probe	Leica Biosystems	Bond polymer refine detection kit	prediluted	0-10% vs. >10%
PDL2	D7U8C	Cell Signalling	Bond polymer refine detection kit	1 to 100	30%

Supplementary Table 3: Availabilities for utilized methods, tools, databases and software

Method/Tool/Software	Version	Available at	Ref.	Notes
HISAT2	2.1.0	http://daehwankimlab.github.io/hisat2/download	58, 59	Alignment method
Genome Analysis Toolkit (GATK) / Mutect	4.1.2.0 / 2.1	https://github.com/broadinstitute/gatk/releases	61, 62	GATK contains Mutect 2.1 for variant discovery
TransVar	2.4.1	https://github.com/zwdzwd/transvar	64	Variant annotator (installed via pip)
MutSig2CV	3.11	https://software.broadinstitute.org/cancer/cga/mutsig	71	Gene level mutation analysis (registration required)
DESeq2	1.30.0	https://bioconductor.org/packages/DESeq2/	91	Differential expression analysis of shRNAs
BLAT	36, standalone	https://users.soe.ucsc.edu/~kent/src/	90	Used for alignment of reads for shRNA screening
NGSCheckMate	1.0.0	https://github.com/parklab/NGSCheckMate	60	Matching QC of tumor/normal pairs
Chromosome Analysis Suite (ChAS)	4.0	https://www.thermofisher.com/de/de/home/life-science/microarray-analysis/microarray-analysis-instruments-software-services/microarray-analysis-software/chromosome-analysis-suite.html	n.a.	Software suite from SNP array manufacturer
ASCAT	2.4.3	https://github.com/Crick-CancerGenomics/ascat	16	Copy number segmentation and sample purity
GISTIC	2.0.23	http://portals.broadinstitute.org/cgi-bin/cancer/publications/pub_paper.cgi?mode=view&paper_id=216&p=t	15	Cohort level SNCA analysis (Matlab runtime required)
Torrent Suite	5.12.0	https://github.com/iontorrent/TS	n.a.	For validation by targeted resequencing (license/measurement device required)
Ion Reporter Software	5.16.0.2	https://www.thermofisher.com/order/catalog/product/4487118#/4487118	n.a.	For validation by targeted resequencing (license/measurement device required)
Integrated Genomics Viewer	2.6.3-2.8.0	http://software.broadinstitute.org/software/igv/download	75	Variant plots in context of their measured reads for visual QC
ProteinPaint	n.a. (web app)	https://pecan.stjude.cloud/proteinpaint	79	Mutation overview plots over protein sequences
Open-Source PyMOL	2.2.0	https://pymol.org/2	81	Mutation overview plots in 3D protein context
GNU parallel	20161222	https://www.gnu.org/software/parallel/	77	Local parallelization of jobs capsuled as bash scripts
samtools	1.10	http://www.htslib.org	76	Sequence file related tasks (manual QC, resorting, indexing)
bedtools	2.27.1	https://bedtools.readthedocs.io	74	QCs
maftools	2.7.41	https://bioconductor.org/packages/maftools	78	TMB plot
picard	2.18.4	https://broadinstitute.github.io/picard/	n.a.	Sequence file related tasks (manual QC, resorting, indexing)
CrossMap	0.4.0	http://crossmap.sourceforge.net	73	Genome coordinates conversion between different assemblies
vcfanno	0.3.0	https://github.com/brentp/vcfanno/releases	70	Quickly combine variant annotation sources

IDEs, general purpose software, runtimes	Version	Available at	Ref.	Notes
MATLAB	R2018a-R2020a	https://www.mathworks.com/pricing-licensing.html?prodcode=ML&intendeduse=edu	n.a.	General purpose analysis software suite (license required)
Microsoft Excel	2016-2019	https://www.microsoft.com/en-us/microsoft-365/excel	n.a.	Spreadsheet software for collecting clinical metadata and presenting analysis results (license required)
Python	2.7 and 3.6	https://www.python.org/	n.a.	General purpose language and runtime (needed by TransVar)
R	3.6.3	https://www.r-project.org/	n.a.	Statistics analysis language and runtime (needed by ASCAT)

Resources/databases	Version	Available at	Ref.	Notes
COSMIC	85	https://cancer.sanger.ac.uk/cosmic	67	Resource database of known somatic mutations
NCBI ClinVar	20180429	https://www.ncbi.nlm.nih.gov/clinvar/	69	Resource database of variants with known clinical significance
gnomAD/ExAC	based on v2	provided via GATK resource pack (file af-only-gnomad.hg38.ensemble.vcf.gz); see https://gatk.broadinstitute.org/hc/en-us/articles/360035890811-Resource-bundle	63	Resource database of known population germline variants, originally based on ExAC
PDBe-KB	2020	https://www.ebi.ac.uk/pdbe/pdbe-kb	80	Used for depicting protein 3D structures in Fig. 1c
NCBI Common Human Variants	20180418	file common_all.vcf.gz from https://www.ncbi.nlm.nih.gov/variation/docs/human_variation_vcf	68	Resource database of common human variants, part of dbSNP build 151
NCBI RefSeq gene models	20190227	provided via TransVar download; file hg38.refseq.gff.gz.transvardb	65	Gene models used for advanced variant annotation, e.g. in the codon frame context
APPRIS	20200122	https://github.com/appris/appris	66	Principal isoforms/transcripts of genes
Human Reference Genome	GRCh38	http://daehwankimlab.github.io/hisat2/download/#h-sapiens	59	Besides the reference genome, this resource contains indexes for sequence alignment via HISAT2; the identical reference genome was utilized for all downstream analyses

Supplementary Table 4: List of used primers

Name	Sequence	Target
sh*IRF4* #1	CCGCCATTCCTCTATTCAAGA	*IRF4*
sh*IRF4* #2	GTGCCATTTCTCAGGGAAGTA	*IRF4*
sh*STAT3* #1	GCCACTTTGGTGTTTCATAAT	*STAT3*
sh*STAT3* #2	GCATAGCCTTTCTGTATTTAA	*STAT3*
