# Supplementary material for: Molecular and functional profiling identifies therapeutically targetable vulnerabilities in plasmablastic lymphoma
Source: Nat Commun. 2021 Aug 31;12:5183. doi: 10.1038/s41467-021-25405-w (PMC8408158; doi:10.1038/s41467-021-25405-w)
Supplement: Supplementary file 3 — Description of Additional Supplementary Files [file 41467_2021_25405_MOESM3_ESM.pdf]

Description of Additional Supplementary Files

Supplementary Data 1: Overview of all primary PBL samples with corresponding clinical and experimental data (IHC/FISH, WES, and SNP).

Supplementary Data 2: Overview of the optimized multistage filter hierarchy showing all variant filter steps in the applied order for calling somatic mutations.

Supplementary Data 3: Cancer gene prediction according to MutSig2CV.

Supplementary Data 4: List of all called somatic variants in 85 primary PBL samples.

Supplementary Data 5: Subcohort comparisons for selected gene sets.

Supplementary Data 6: Mutations revealed by targeted resequencing of the selected cancer candidate genes TP53, NRAS, KRAS, BRAF, STAT3, and TET2.

Supplementary Data 7: List of recurrent somatic copy number alterations in 82 primary PBL samples.

Supplementary Data 8: Gene level view of copy number alterations in 82 primary PBL samples and the PBL-1 cell line.

Supplementary Data 9: Survival analysis for preselected variables using log-rank tests and unbiased univariate screening of all available biological covariates using Cox proportional hazard models.

Supplementary Data 10: Depletion ratios of all used shRNAs in the performed shRNA screen.
