# Supplementary material for: Molecular and functional profiling identifies therapeutically targetable vulnerabilities in plasmablastic lymphoma
Source: Nat Commun. 2021 Aug 31;12:5183. doi: 10.1038/s41467-021-25405-w (PMC8408158; doi:10.1038/s41467-021-25405-w)
Supplement: Supplementary file 14 — Reporting summary [file 41467_2021_25405_MOESM14_ESM.pdf]

nature research

Corresponding author(s): Georg Lenz

Last updated by author(s): Jul 15, 2021

# Reporting Summary

Nature Research wishes to improve the reproducibility of the work that we publish. This form provides structure for consistency and transparency in reporting. For further information on Nature Research policies, see our Editorial Policies and the Editorial Policy Checklist.

## Statistics

For all statistical analyses, confirm that the following items are present in the figure legend, table legend, main text, or Methods section.

| n/a | Confirmed | |
|---|---|---|
| ☐ | ☒ | The exact sample size ($n$) for each experimental group/condition, given as a discrete number and unit of measurement |
| ☐ | ☒ | A statement on whether measurements were taken from distinct samples or whether the same sample was measured repeatedly |
| ☐ | ☒ | The statistical test(s) used AND whether they are one- or two-sided<br>*Only common tests should be described solely by name; describe more complex techniques in the Methods section.* |
| ☐ | ☒ | A description of all covariates tested |
| ☐ | ☒ | A description of any assumptions or corrections, such as tests of normality and adjustment for multiple comparisons |
| ☐ | ☒ | A full description of the statistical parameters including central tendency (e.g. means) or other basic estimates (e.g. regression coefficient) AND variation (e.g. standard deviation) or associated estimates of uncertainty (e.g. confidence intervals) |
| ☐ | ☒ | For null hypothesis testing, the test statistic (e.g. $F$, $t$, $r$) with confidence intervals, effect sizes, degrees of freedom and $P$ value noted<br>*Give P values as exact values whenever suitable.* |
| ☒ | ☐ | For Bayesian analysis, information on the choice of priors and Markov chain Monte Carlo settings |
| ☒ | ☐ | For hierarchical and complex designs, identification of the appropriate level for tests and full reporting of outcomes |
| ☒ | ☐ | Estimates of effect sizes (e.g. Cohen's $d$, Pearson's $r$), indicating how they were calculated |

*Our web collection on statistics for biologists contains articles on many of the points above.*

## Software and code

Policy information about availability of computer code

| | |
|---|---|
| Data collection | Data from high-throughput sequencing (FASTQ files) and SNP microarray measurements (CEL files) were directly processed in their raw form with our analysis pipeline (see Supplementary Figure 3). Metadata and data were collected and stored in Microsoft Excel (v2016-2019) tables. |
| Data analysis | We used these software programs and packages (in lexical order): ASCAT 2.4.3, bedtools 2.27.1, Chromosome Analysis Suite (ChAS) 4.0, CrossMap 0.4.0, DESeq2 1.30.0, BLAT 36 standalone, Genome Analysis Toolkit (GATK) 4.1.2.0 with Mutect 2.1, GISTIC 2.0.23, GNU parallel 20161222, HISAT2 2.1.0, Integrated Genomics Viewer 2.6.3-2.8.0, Ion Reporter Software 5.16.0.2, maftools 2.7.41, MathWorks MATLAB R2018a-R2020a, Microsoft Excel 2016-2019, MutSig2CV 3.11, NGSCheckMate 1.0.0, Open-Source PyMOL 2.2.0, picard 2.18.4, ProteinPaint web app, Python 2.7 and 3.6, R 3.6.3, samtools 1.10, Torrent Suite 5.12.0, TransVar 2.4.1, and vcfanno 0.3.0.<br>See Supplementary Figure 3 for a schematic overview of main analyses. Detailed descriptions of our analyses are provided in methods and Supplementary Information. Supplementary Table 3 provides more details on tool availabilities including download URLs. |

For manuscripts utilizing custom algorithms or software that are central to the research but not yet described in published literature, software must be made available to editors and reviewers. We strongly encourage code deposition in a community repository (e.g. GitHub). See the Nature Research guidelines for submitting code & software for further information.

## Data

Policy information about availability of data

All manuscripts must include a data availability statement. This statement should provide the following information, where applicable:

- Accession codes, unique identifiers, or web links for publicly available datasets
- A list of figures that have associated raw data
- A description of any restrictions on data availability

The whole exome sequencing data EGAD00001006400 [https://ega-archive.org/datasets/EGAD00001006400] and SNP microarray data EGAD00010001978 [https://

ega-archive.org/datasets/EGAD00010001978] generated in this study have been deposited in the European Genome-phenome Archive (EGA) under study accession EGAS00001004659 [https://ega-archive.org/studies/EGAS00001004659]. These data are available under restricted access for German data privacy laws; access can be obtained via the associated data access committee EGAC00001001735 [https://ega-archive.org/dacs/EGAC00001001735]. The processed somatic mutations and copy number aberrations as well as clinical metadata and figure raw data are provided in respective Supplementary Data items or the Source Data file.

The following public data sources were used in this study: The human reference genome from the Genome Reference Consortium (GRCh38) in its pre-indexed form for alignment with HISAT2 [http://daehwankimlab.github.io/hisat2/download/#h-sapiens], the Catalogue Of Somatic Mutations In Cancer (COSMIC, v85) [https://cancer.sanger.ac.uk/cosmic], the NCBI database of common human variants (based on dbSNP build 151, version 2018-04) [https://www.ncbi.nlm.nih.gov/variation/docs/human_variation_vcf]), NCBI ClinVar (version 2018-04) [https://www.ncbi.nlm.nih.gov/clinvar/], gnomAD/ExAC germline variants as provided in the file af-only-gnomad.hg38.ensemble.vcf.gz of the GATK resource bundle [originally accessed via ftp.broadinstitute.org/bundle, but since moved by the Broad Institute to Google cloud bucket; see https://gatk.broadinstitute.org/hc/en-us/articles/360035890811-Resource-bundle for access information], the PDBe-KB for 3D protein information [https://www.ebi.ac.uk/pdbe/pdbe-kb] and the principal splice isoforms database (APPRIS, version 2020-01-22) [https://github.com/appris/appris].

# Field-specific reporting

Please select the one below that is the best fit for your research. If you are not sure, read the appropriate sections before making your selection.

- [x] Life sciences
- [ ] Behavioural & social sciences
- [ ] Ecological, evolutionary & environmental sciences

For a reference copy of the document with all sections, see nature.com/documents/nr-reporting-summary-flat.pdf

# Life sciences study design

All studies must disclose on these points even when the disclosure is negative.

**Sample size**

Within this exploratory retrospective study we aimed to obtain a better understanding of the molecular pathogenesis of plasmablastic lymphoma (PBL) which represents an extremely rare disease. To this end, the maximum number of available primary patient samples were collected in different European centers. We were able to collect 96 primary PBL samples for further analyses. Therefore, no sample size or power calculation were necessary and accordingly performed. For composition of our study cohort, see Supplementary Figure 1. One plasmablastic cell line, PBL-1, and a panel of 7 DLBCL cell lines were used for functional validation (see Figure 4).

**Data exclusions**

Our criteria were pre-established for data exclusion:
1. Obtain sufficient DNA to analyze samples by WES, targeted resequencing and Oncoscan.
2. Obtain sufficient quality control in these analyses.
3. Obtain sufficient primary material to build a tissue microarray (TMA).

Accordingly, for 89 primary PBL samples we could extract sufficient DNA to perform WES. 4 samples were excluded after alignment and coverage QC (outliers with too few mapped reads, relative to the distribution of mapped reads in all samples). For 54 PBL cases we could extract sufficient DNA to perform targeted resequencing (>20 ng, pre-established threshold as per Thermo-Fisher technical documents). For 86 PBL samples we obtained sufficient DNA to perform Oncoscan (>80 ng, pre-established threshold as per Thermo-Fisher technical documents). 4 samples were excluded after ASCAT QC (pre-established binary quality estimate in the ASCAT algorithm). 68 PBL samples were eligible for TMA construction: for these samples we uniformly performed IHC and FISH.

**Replication**

For genomic analysis, no replication was performed. Instead, we utilized statistical measures including estimation of false positive rates to assess reproducibility of recurrent findings on cohort level over independent subjects.

Constructed TMA contained 3 replicates of each tumor sample for IHC/FISH. In general, replication for TMA experiments was successful. In case that a single replicate failed we analyzed the remaining two samples.

All functional experiments were performed at least twice. Precise numbers of repetitions are indicated for each experiment (Figure 4, Supplementary Figure 10).

**Randomization**

As this is a retrospective exploratory study randomization was not applicable.

**Blinding**

As this is a retrospective exploratory study blinding was not applicable.

# Reporting for specific materials, systems and methods

We require information from authors about some types of materials, experimental systems and methods used in many studies. Here, indicate whether each material, system or method listed is relevant to your study. If you are not sure if a list item applies to your research, read the appropriate section before selecting a response.

## Materials & experimental systems

| n/a | Involved in the study | |
|---|---|---|
| ☐ | ☒ | Antibodies |
| ☐ | ☒ | Eukaryotic cell lines |
| ☒ | ☐ | Palaeontology and archaeology |
| ☒ | ☐ | Animals and other organisms |
| ☐ | ☒ | Human research participants |
| ☒ | ☐ | Clinical data |
| ☒ | ☐ | Dual use research of concern |

## Methods

| n/a | Involved in the study | |
|---|---|---|
| ☒ | ☐ | ChIP-seq |
| ☒ | ☐ | Flow cytometry |
| ☒ | ☐ | MRI-based neuroimaging |

# Antibodies

Antibodies used

Used antibodies for immunohistochemistry are provided in Supplementary Table 2.
Used antibodies for Western blotting: anti IRF4 Cell Signaling #4964, anti STAT3 Cell Signaling #9139, anti pSTAT3(Tyr705) Cell Signaling #9145, anti alpha-Tublin Sigma #T9026.

Validation

Indicated antibodies are all validated for use in human tissue/cell lines (see datasheets on homepages of respective manufracturers): anti IRF4 Cell Signaling #4964 (https://www.cellsignal.com/products/primary-antibodies/irf-4-antibody/4964), anti STAT3 Cell Signaling #9139 (https://www.cellsignal.com/products/primary-antibodies/stat3-124h6-mouse-mab/9139), anti pSTAT3(Tyr705) Cell Signaling #9145 (https://www.cellsignal.com/products/primary-antibodies/phospho-stat3-tyr705-d3a7-xp-rabbit-mab/9145), anti alpha-Tublin Sigma #T9026 (https://www.sigmaaldrich.com/DE/de/product/sigma/t9026).

# Eukaryotic cell lines

Policy information about cell lines

Cell line source(s)

PBL-1 cell line was derived from an HIV-positive PBL patient and established in the Katano laboratory in Tokyo (Mine et al. 2017). All DLBCL cell lines Karpas 422 (K422), HT, DB, OCI-Ly1 , WSU-DLCL2, OCI-Ly10, and BJAB were obtained from the Louis Staudt laboratory (NCI Bethesda).

Authentication

STR profiling was performed for OCI-Ly1 and OCI-Ly10. K422, HT, DB, WSU-DLCL2, and BJAB were authenticated using SNP profiling.
PBL-1 could not be authenticated but was obtained from the laboratory that created the cell line. We have confirmed its typical immunophenotype by immunhistochemical staining and detected common genetic lesions frequently detectable in primary PBL patient samples (Supplementary Figure 1, Supplementary Data 4 and 8).

Mycoplasma contamination

All cell lines were tested negative for mycoplasma contamination.

Commonly misidentified lines
(See ICLAC register)

None.

# Human research participants

Policy information about studies involving human research participants

Population characteristics

96 formalin-fixed paraffin embedded (FFPE) human PBL samples were collected from the University Hospitals in Münster, Kiel, Würzburg, Berlin, Basel, Glasgow, Tübingen, the Robert-Bosch Hospital in Stuttgart, and the Germans Trias i Pujol Hospital, University Hospital of Bellvitge, Hospital Vall d'Hebron and Hospital del Mar in Barcelona, and Hospital Gregorio Marañón in Madrid. Available clinical data are summarized in Supplementary Table 1. For 49 patients we obtained survival data as summarized in Fig. 3, Supplementary Data 1 and 9.

Recruitment

Clinical data were retrospectively retrieved from clinical records.

Ethics oversight

Alive patients were asked for informed consent as requested by the ethical committees. Full approval was obtained by the institutional ethics review boards of the University Hospitals in Tübingen, Glasgow, and Basel and the Germans Trias i Pujol Hospital in Barcelona, in accordance with the Declaration of Helsinki.

Note that full information on the approval of the study protocol must also be provided in the manuscript.
